# Practical Opportunities for Biopsychosocial Education Through Strategic Interprofessional Experiences in Integrated Primary Care

**DOI:** 10.3389/fpsyt.2021.693729

**Published:** 2021-09-16

**Authors:** Jennifer S. Funderburk, Julie Gass, Robyn L. Shepardson, Luke D. Mitzel, Katherine A. Buckheit

**Affiliations:** ^1^Veterans Affairs Center for Integrated Healthcare, Syracuse VA Medical Center, Syracuse, NY, United States; ^2^Department of Psychology, Syracuse University, Syracuse, NY, United States; ^3^Department of Psychiatry, University of Rochester Medical Center, Rochester, NY, United States; ^4^Veterans Affairs Center for Integrated Healthcare, Western New York VA Healthcare System, Buffalo, NY, United States; ^5^Department of Psychology, University at Buffalo, State University of New York, Buffalo, NY, United States

**Keywords:** interprofessional education, biopsychosocial, integrated primary care, shared medical appointment, huddle

## Abstract

Even with the expansion of primary care teams to include behavioral health and other providers from a range of disciplines, providers are regularly challenged to deliver care that adequately addresses the complex array of biopsychosocial factors underlying the patient's presenting concern. The limits of expertise, the ever-changing shifts in evidence-based practices, and the difficulties of interprofessional teamwork contribute to the challenge. In this article, we discuss the opportunity to leverage the interprofessional team-based care activities within integrated primary care settings as interactive educational opportunities to build competencies in biopsychosocial care among primary care team members. We argue that this approach to learning while providing direct patient care not only facilitates new provider knowledge and skills, but also provides a venue to enhance team processes that are key to delivering integrated biopsychosocial care to patients. We provide three case examples of how to utilize strategic planning within specific team-based care activities common in integrated primary care settings—shared medical appointments, conjoint appointments, and team huddles—to facilitate educational objectives.

## Introduction

Providing whole-person care that addresses the complex array of biopsychosocial factors contributing to patients' health concerns is a perpetual challenge in primary care (PC). For instance, a patient can present with psychological distress from depression and/or food insecurity, which can drastically impact diabetes management. These factors can, in turn, also contribute to the patient's decisions regarding engagement in certain health behaviors, such drinking alcohol, that may negatively impact chronic medical conditions. This example clarifies why primary care providers (PCPs) have been encouraged to switch from traditional models of focusing only on biological factors of health toward the biopsychosocial model, which recognizes biological, psychological, and social factors and their interactions that contribute to health and well-being (1). However, the limits of expertise for any one PC team member are stretched by patients presenting with a range of medical concerns that are often further complicated by psychological distress and/or unmet social needs (2). In addition, PC team members are frequently required to rapidly shift clinical practices to stay up-to-date with the latest research (3). For instance, research has demonstrated the value of using psychological treatments for several health concerns [e.g., insomnia (4), chronic pain (5)]. Yet, PCPs struggle to embrace new clinical practices and engage patients in these new treatments (6). The shift toward team-based care within PC settings (7) is intended to help address these gaps and improve the quality of patient care by adding members with complementary and specialized skillsets to teams, such as behavioral health providers (BHPs; e.g., psychologists, social workers) (8). These additional team members have the skills to support the PC team in improving their approach to talking to and directly helping patients.

However, simply embedding additional providers in PC does not yield instant success in overcoming barriers to delivery of biopsychosocial care. High-quality, patient-centered care that recognizes the biopsychosocial contributions to patients' presenting concerns will not be delivered unless teams move beyond a referral model, in which the factors contributing to disease are compartmentalized and handled separately by different providers. Instead, all providers need to embrace the biopsychosocial model and work cohesively as a team, and collaboratively with the patient, to recognize, support, and implement strategies jointly targeting biopsychosocial factors. The purpose of this article is to discuss strategies to leverage interprofessional clinical experiences within integrated PC settings to facilitate interactive, biopsychosocial education for providers that ultimately improves patient care. We present three approaches using study protocols currently being piloted.

### Rationale

PC education begins in healthcare training programs and is supplemented by continuing education. Many of these educational opportunities are provider- or discipline-specific, use formal learning approaches [i.e., organized didactic learning events (9, 10)], require time separate from direct patient care, and result in small-to-moderate changes in provider behavior (11–13). Given these limitations, there is a need for creative solutions to assist PC team members, especially within the context of biopsychosocial approaches to care.

Building on the framework of social learning theory (14), leveraging the presence of the interprofessional team can provide an innovative way to achieve interactive education in which skills can be learned via observation and modeling from others within the team while delivering patient care. This allows multiple team members from a range of disciplines to learn through informal and experiential interprofessional education during direct patient care (15) without requiring providers to carve out additional time. Interprofessional team education with trainees has been shown to increase knowledge, teamwork, satisfaction, and improve delivery of care to patients (16).

This approach of learning through team-based care activities also allows for the PC team members to not only gain new knowledge on specific presenting concerns, but also further develop their skills in the team processes that are key to collaboratively providing integrated biopsychosocial care to patients. Salas and colleagues (17) have identified several essential elements that underlie successful teamwork, such as communication, coordination, and cooperation. Engaging PC teams in specific activities that require team members to work together in a structured way provides real-world opportunities to improve all these skills during clinical activities.

### Types of Team-Based Activities That Can Serve as Educational Opportunities

Several team-based direct patient care activities that already occur within integrated PC settings can be strategically infused with interactive, interprofessional, biopsychosocial education. Examples include: shared medical appointments (SMAs; also known as group medical visits) in which the PCP and other members of the team such as the embedded BHP meet with a group of patients with a common presenting concern [e.g., (18)]; team huddles, “a brief, frequent form of structured communication among members of the PC team” to discuss patient care and maximize efficiency (19); and conjoint appointments in which two providers (e.g., PCP and the embedded BHP) meet jointly with a patient to discuss a specific concern (20). All three team-based examples are patient-care activities that can also facilitate education. These activities are well-suited for, and enhanced by, a biopsychosocial lens as they often consider a range of biomedical, psychological, and social factors relevant to patients and utilize a range of interventions including psychoeducation, medication management, and evidence-based behavioral strategies (21).

### Strategic Interprofessional Education

Strategic planning is necessary to optimize the interprofessional educational yield of these team-based care activities, as social learning theory suggests the activities need to not only include observation/modeling, but also attend to cognitive processes (i.e., motivation, attention, retainment, and reproduction) to maximize learning (14). The topic of the team-based care activity needs to be relevant and meaningful for all team members involved to help motivate learning (22). The specific educational objectives should be identified ahead of time. As our protocols detailed below highlight, leveraging activities that are already a part of providers' daily provision of direct patient care and identifying specific educational objectives that are of interest to providers increases the direct relevance of the information, which improves adult learning (23). In addition, the team-based activity needs to ensure interactive learning can take place through either observation and/or simulation. The team-based activity also needs to go beyond shared learning or working in tandem to engage providers in interprofessional collaboration for informal and experiential learning to take place (22) while simultaneously attending to interprofessional team processes, such as role clarity and communication. Therefore, specific strategies to encourage the team to attend to the material one another are sharing and work together toward shared objectives are key. Finally, the team-based activity needs to provide opportunities for the team members to reproduce the learned information. The three team-based activity protocols below highlight strategies to integrate interprofessional biopsychosocial education and clinical care into routine PC practice.

## Three Different Approaches/Protocols

### Interprofessional Structured Shared Medical Appointment for Chronic Pain

Chronic pain is highly prevalent in PC, yet access to specialty pain clinics is limited (24), leaving most chronic pain patients [i.e., 52%; (25)] treated by PCPs. However, PCPs receive little education regarding the treatment of chronic pain, particularly from a biopsychosocial (compared to biomedical) perspective (26, 27). PCPs often report feeling the least confident in their ability to manage chronic pain patients compared to other providers [e.g., specialty pain physicians; (25)] and that treatment of chronic pain is a substantial source of dissatisfaction (28).

Less formal experiential education strategies, particularly those incorporating interprofessional consultation, improve quality of care (29) and enhance knowledge (16). If implemented effectively, experiential interprofessional education strategies may help improve management of chronic pain specifically (26). Based on previous research demonstrating the efficacy of SMAs for chronic pain (30, 31), we examined an SMA to address chronic pain as a clinical demonstration in a United States Veterans Health Administration (VHA) PC clinic. This was a 5-session, closed, ~75-min group visit delivered across 7 weeks for patients (*n* = 6) with musculoskeletal chronic pain. The SMA content focused on two evidence-based approaches: medication education and management delivered by a clinical pharmacist and PCP (32) and Cognitive-Behavioral Therapy for Chronic Pain (CBT-CP) delivered by a BHP, which improves pain intensity and pain self-efficacy (5).

We incorporated additional structure into the SMA to maximize interprofessional informal and experiential learning among the PCP, clinical pharmacist, and BHP on the provision of evidence-based biopsychosocial chronic pain management. The educational objectives were to improve the PCP's knowledge and use of biopsychosocial approaches to pain management and to improve their knowledge of pain medication management strategies. A 25-min team briefing was held prior to the initial SMA appointment to ensure all team members knew one another and their specific role in the SMA, as well as provide an opportunity for members to review aspects of evidence-based chronic pain management together. The PCP and BHP co-lead the introduction to some material, which allowed the PCP to observe the BHP presenting the biopsychosocial model of pain (SMA appointment 1) and cognitive aspects of CBT (SMA appointment 4) to promote the PCP's experiential learning of these key aspects of care. The clinical pharmacist was asked to review each SMA patient's medical chart and provide recommendations to the PCP prior to the first and fourth SMA visit. These recommendations were discussed among providers, thus allowing for experiential learning of evidence-based pain medication management strategies, a specialty of clinical pharmacists. Finally, we asked the BHP to engage in measurement-based care and provide that information and behavioral recommendations to the PCP, which allowed the PCP to understand the impact/intensity of the patient's pain and develop a basic understanding of CBT-CP approach. Preliminary feedback using follow-up qualitative interviews with 2 PCPs and 1 BHP on this innovative, strategic approach revealed that the PCPs reported an improved understanding of the biopsychosocial model, work satisfaction, and confidence in caring for patients with chronic pain.

### Team Huddles

PC is critical to suicide prevention, as a majority of patients who die by suicide were seen in PC in the month prior to suicide (33). The American National Action Alliance for Suicide has developed clinical practice guidelines (CPGs) to assist PCPs in providing evidence-based care for patients at-risk for suicide (34). A team-based, biopsychosocial approach is particularly important in applying the first step of the CPGs, determining that a patient is at-risk for suicide, as PC team members can help identify various biological (e.g., chronic pain), psychological (e.g., depressive symptoms), and/or social (e.g., job loss) risk factors for suicide. However, consistent provision of care that is concordant with CPGs for suicide prevention remains a critical concern (13, 35, 36). This may be due in part to ineffective formats for educating providers, as didactic education and passive dissemination do not sufficiently improve knowledge of and adherence to CPGs (13). Another potential barrier is that patient openness to sharing suicide-related information is influenced by how PC team members ask risk assessment questions and facilitate rapport (37). Therefore, efforts to educate providers on suicide prevention CPGs need to attend to both approach and content; that is, delivering highly adherent, evidence-based care in a patient-centered way. This is a challenge for healthcare professionals with little or ineffective training on this topic (38).

Team huddles offer an opportunity to strategically address these challenges. Several key functions of team huddles can help to improve patient care, including reviewing and planning for upcoming patients; improving team communication and coordination efforts; and increasing shared awareness of team members' roles and tasks (39). Research supports these benefits of huddles, such that PC team members who attended huddles reported higher scores on teamwork, decision-making, and psychological safety within the team compared to those who did not attend huddles (39). Thus, if used consistently by all team members, huddles can serve as a powerful tool to improve team functioning and patient care. In practice, however, strategic planning is helpful to facilitate optimal interprofessional education in huddles and overcome barriers such as lack of regular attendance (7, 39).

Our team has developed Team Education for Adopting Change in Healthcare (TEACH), a series of four brief team meetings that mimic a huddle format, to improve suicide prevention practices within integrated PC. All members of the PC team are involved in TEACH meetings, including the embedded BHP. TEACH incorporates interactive education with experiential learning components using a simulation strategy (40) to help improve knowledge of and familiarity with CPGs as well as team briefing and debriefing, which has been found in prior research to improve team processes (41–43). As shown in Table 1, biopsychosocial care is a primary educational objective that is reinforced during each meeting by reviewing which team members should assess or provide treatment for biomedical (e.g., PCP prescribes medication), psychological (e.g., BHP develops safety plan with the patient), and social (e.g., social worker connects patient to housing resources) concerns that may contribute to suicide risk. Other educational objectives are to improve team knowledge of the CPGs and team processes to ensure high quality delivery. To increase fidelity to and the impact of TEACH, the four team meetings are dispersed across 12 weeks and occur within the natural work environment (including virtual care if applicable) (44). TEACH is currently being piloted within 2 VHA integrated PC clinics, with 4 teams receiving TEACH and 4 teams continuing to receive standard suicide prevention support. Data will be collected from team members, and the electronic medical record to preliminarily examine feasibility and acceptability.

**Table 1:** Goals of four meetings in the TEACH intervention.

**Meeting #**	**Type**	**Content of meeting**
1	Overview	• Orientation to TEACH meeting format and goals • Discuss role of entire primary care team and team process as review the clinical practice guidelines for suicide
2	Team briefing part 1	• Identify roles of team and how communication works between providers when encountering different types of patients, who report suicidal ideation
3	Team briefing part 2	• Simulate delivering clinical practice guideline-concordant care at an upcoming at-risk patient's appointment
4	De-briefing	• Review how the process went with a previous at-risk patient and problem solve issues

### Conjoint Appointments

Cardiovascular diseases (CVDs) are among the most common (~40–60%) and costly health concerns among United States military Veterans (45, 46), and the monitoring of and prevention efforts for CVDs tends to occur in PC settings (47). CVDs are influenced by a number of behavioral factors, such as drinking alcohol at risky levels (48–51) and behaviors which are affected by psychosocial factors (e.g., low motivation to change behavior). PCPs who feel uncomfortable addressing the psychosocial aspects of CVD are less likely to fully address biopsychosocial concerns like risky drinking (52). Many PCPs (68%) do not have prior experience with motivational interventions and report lower confidence than BHPs at strengthening patient motivation (53). On the other hand, a BHP alone may not be trained/able to address all the biological aspects of smoking/drinking, particularly when they occur in the context of comorbid and complex health conditions such as CVD (54). Medical comorbidities can be important motivators for patients who engage in risky alcohol or tobacco use (55). Thus, if BHPs are not routinely discussing the connection between health and behavior, there may be missed opportunities to inspire change.

Research has found that observing/shadowing providers in action improves a learner's ability to provide comprehensive, biopsychosocial care in the future (56). Experiential learning also improves interprofessional awareness and team functioning, as providers gain appreciation for their colleagues' expertise (57), and solidifies non-experience driven learning. Thus, we have developed and are piloting a *conjoint appointment* protocol called Cardiovascular disease and substance Risk Education—Patient Aligned Care Team (CARE-PACT) where a PCP and BHP meet dually with a patient to discuss the patient's smoking/risky drinking within the context of their diagnosed CVD. In bringing together providers with expertise in differing areas of the biopsychosocial spectrum, conjoint appointments are an excellent approach for patients who have mental/behavioral health concerns related to medical concerns (20, 58, 59) and also provide an opportunity for providers to demonstrate and acquire biopsychosocial skills through collaboration with providers from a different training background.

**Figure 1:**
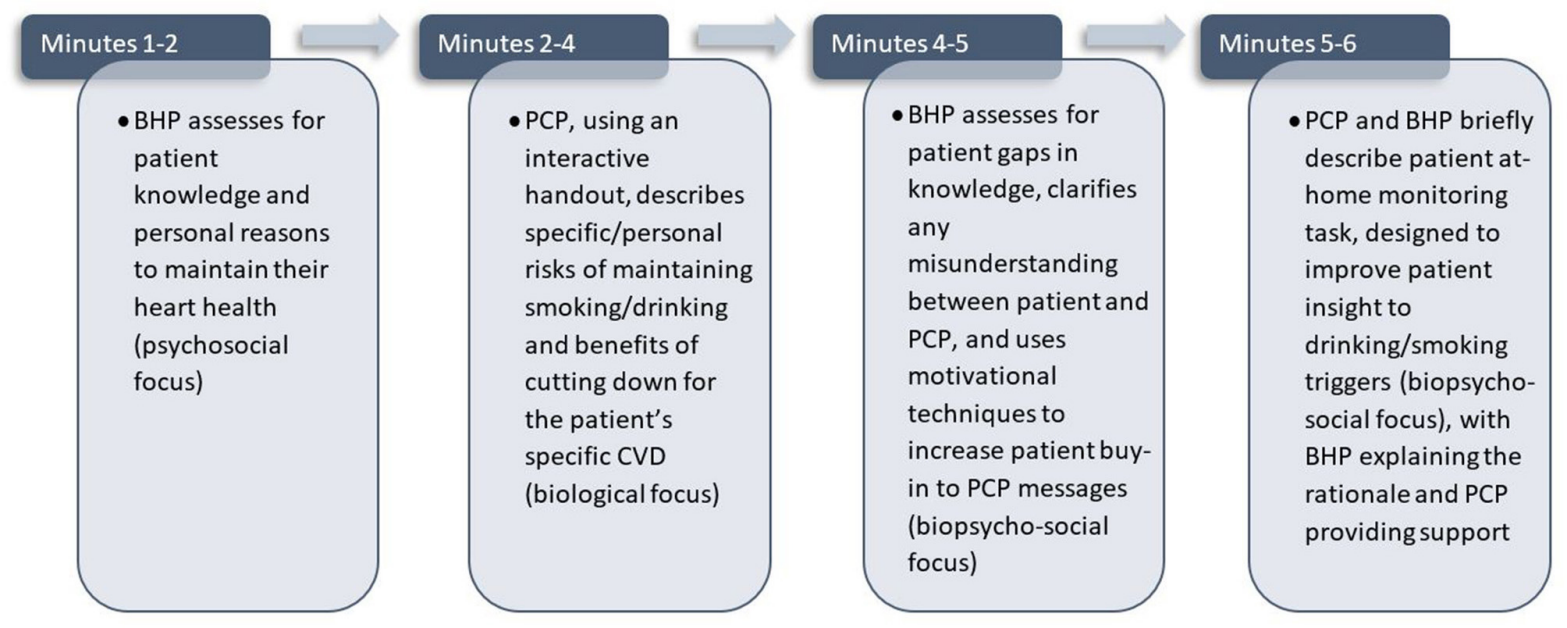
Minute-by-minute description of activities conducted during the 6-min encounter when delivering CARE-PACT.

In CARE-PACT, the PCP and BHP each have specific roles and content areas to share with the patient during a brief 5–7 min encounter. As outlined within Figure 1, the BHP uses motivational interviewing approaches (60) to evaluate patient motivational factors, assess for understanding, and increase patient-buy in, thus providing the opportunity for PCPs to learn. The PCP addresses the patient's personal risks and potential benefits of changing smoking/drinking given their specific CVD, thus providing the opportunity for the BHP to learn more about biological complexities associated with smoking/drinking. The conjoint appointment ends with the patient having the option to follow-up with the BHP. CARE-PACT is currently being piloted in an open trial in two VHA PC clinics, where 4 PCPs and their embedded BHP will deliver CARE-PACT to 15 PC patients with cardiovascular disease who engage in at-risk alcohol use/smoking. Following the intervention, patients and providers will provide feedback on acceptability and feasibility.

## Discussion

These three protocols demonstrate how team-based care activities with empirical support for improving direct patient care, such as SMAs (30, 31), can be strategically structured to provide opportunities for biopsychosocial education of PC team members. Although the specific protocols shared in this article are still undergoing formal evaluation as venues for interprofessional education, the strategic education provided within these team-based care activities has the potential to improve not only provider understanding and utilization of patient-centered biopsychosocial approaches to care, but also teamwork processes in the relational aspects of care delivery [e.g., shared mental models; (17)] by giving team members additional opportunities to collaborate. These team-based care activities also pragmatically leverage real-world clinical care activities to bridge conceptual gaps that can only be addressed through interdisciplinary collaboration. Observations from this initial pilot work suggest that these experiences are perceived as rewarding by providers and may also help decrease provider burnout by offering variety in daily activities. However, future research is needed to fully understand the educational value of these activities on their own or in comparison to one another as well as continue to identify the benefits of these activities to patient care. Therefore, it is difficult to recommend one approach over another at this time.

All of these case examples were designed to be delivered with all team members being in-person, but the advent of the COVID-19 pandemic has caused a shift toward greater utilization of virtual formats for patients and employees. This shift is likely to remain beyond the current pandemic, as telehealth and telework offer advantages in overcoming scheduling issues as well as sustainability. Existing research suggests that virtual interactive learning methods can still be effective and result in similar educational gains as in-person (61, 62); however, future research would need to determine if other strategies need to be considered to achieve success in provider education via virtual platforms. Similarly, there have been advances in understanding how to navigate the ethical considerations associated with an integrated team approach to patient care (63, 64); however, continued attention to the ethical considerations within these contexts is also necessary.

## Author Contributions

All authors listed have made a substantial, direct and intellectual contribution to the work, and approved it for publication.

## Funding

JF's pilot work on TEACH was supported by the American Foundation for Suicide Prevention (SRG-0-064-19). JG (grant K2 HX002610) and RS (grant IK2 HX002107), are supported by the Department of Veterans Affairs, Veterans Health Administration, Health Services Research and Development Service (HSR&D) Career Development Awards-2. LM and KB are supported by the Department of Veterans Affairs, Office of Academic Affiliations, Advanced Fellowship Program in Mental Illness Research and Treatment. This study was supported with resources and the use of facilities at the VA Center for Integrated Healthcare. The funding sources had no role in the study design; collection, analysis, and interpretation of data; writing of the manuscript; or decision to submit the manuscript for publication.

## Author Disclaimer

The views expressed in this article are those of the authors and do not reflect the official position or policy of the Department of Veterans Affairs or the United States Government.

## Conflict of Interest

The authors declare that the research was conducted in the absence of any commercial or financial relationships that could be construed as a potential conflict of interest.

## Publisher's Note

All claims expressed in this article are solely those of the authors and do not necessarily represent those of their affiliated organizations, or those of the publisher, the editors and the reviewers. Any product that may be evaluated in this article, or claim that may be made by its manufacturer, is not guaranteed or endorsed by the publisher.
